# Occult invasive group A streptococcal infection with rapid progression to pediatric intraorbital abscess: a case report

**DOI:** 10.1186/s12887-026-06717-6

**Published:** 2026-03-12

**Authors:** Yingguang Liu, Yuqin Chu, Na Zhang, Chao Wang, Jingjing Liu, Nan Wu, Pingping Zhang

**Affiliations:** 1 Department of Pediatrics, Department of Neonatology, First Central Hospital Affiliated to Nankai University, Tianjin First Central Hospital, Tianjin, 300192 China; 2 Department of Otorhinolaryngology Head and Neck Surgery, Tianjin First Central Hospital, Tianjin, 300192 China; 3 Department of Pharmacy, Tianjin First Central Hospital, Tianjin, 300192 China; 4 Department of Gynecology, Tianjin First Central Hospital, Tianjin, 300192 China; 5 Key Medical Cultivation Discipline of Tianjin (Otolaryngology), Tianjin, 300192 China; 6 Department of Neurosurgery, Tianjin Children’s Hospital (Children’s Hospital of Tianjin University), Tianjin, 300134 China

**Keywords:** Invasive Group A Streptococcus, Intraorbital Abscess, Pediatric, Sinusitis, Multidisciplinary Team, Case Report

## Abstract

**Background:**

Invasive Group A Streptococcal (iGAS) infection is relatively rare in pediatric upper respiratory tract infections, but it can breach anatomical barriers to cause deep abscesses. However, iGAS-induced rapid development of intraorbital abscess in children is extremely uncommon. Due to its high occult nature, it is easily overlooked, which may lead to permanent visual loss or even death.

**Case description:**

A 6-year-old girl was admitted to the hospital with “rhinorrhea and cough for 1 week, accompanied by fever for 2 days”. Upon admission, the pediatrician diagnosed acute tonsillitis and administered amoxicillin-clavulanate potassium for anti-infective treatment. Four hours after admission, the girl developed right eyelid edema and conjunctival congestion. Six hours after admission, obvious proptosis and diminished direct light reflex were noted in the right eye, and the pediatrician immediately initiated a multidisciplinary team (MDT) consultation. Based on the combined findings of orbital computed tomography (CT) and paranasal sinus magnetic resonance imaging (MRI), MDT confirmed the girl to have sinusitis complicated by orbital cellulitis, and that her condition had progressed to the rapidly progressive stage of intraorbital abscess. Subsequent results of blood culture and next-generation sequencing (NGS) both indicated GAS infection. After a comprehensive evaluation of all clinical indicators, the MDT formulated a treatment plan involving endoscopic sinus surgery combined with orbital abscess incision and drainage via a supraorbital eyebrow approach. The girl received 3 weeks of postoperative anti-infective therapy, with her inflammatory markers returning to normal and visual acuity restored. No recurrence was observed during the follow-up examination 6 months later.

**Conclusions:**

GAS could break through the lamina papyracea in a highly insidious manner, causing invasive infection of orbital tissues, followed by rapid progression of the infection. Clinical diagnosis and treatment emphasizes the rapid response of the MDT consultation. When the child presents with rapid progression of ocular symptoms, it is recommended to escalate the anti-infective regimen and perform early surgical drainage to improve prognosis. This case provides an important reference for the clinical diagnosis and treatment of severe orbital infections caused by iGAS in children.

## Background

Invasive Group A Streptococcal (iGAS) infection refers to a local or systemic infection caused by Group A Streptococcus that breaches the host’s skin and mucosal barriers and invades normally sterile tissues, organs, or body fluids [1, 2]. GAS is a common pathogen of pediatric upper respiratory tract infections. Due to the underdeveloped immune and barrier functions in children, it easily spreads to the adjacent paranasal sinuses [3]. But the process of GAS breaching anatomical barriers and invading the orbit is characterized by poor specific manifestations and a highly occult nature [4]. It is clinically rare with few reported cases, and carries a high risk of misdiagnosis.

The medial wall of the pediatric ethmoid sinus serves as the lateral wall of the nasal cavity and also the medial orbital wall, which is also known as the lamina papyracea [5]. The lamina papyracea is extremely thin with a thickness of only 0.2–0.5 mm, and the bony plate is incompletely ossified in childhood, with natural small foramina or fissures [6]. GAS invades orbital tissues by traversing this barrier, which constitutes an invasive infection accompanied by orbital complications. According to the Chandler classification [7], orbital complications can be divided into 5 stages: Preseptal Cellulitis, Orbital Cellulitis, Subperiosteal Abscess, Intraorbital Abscess, and Cavernous Sinus Thrombosis. Once iGAS infection progresses to the stage of intraorbital abscess, it could result in irreversible visual impairment and even life-threatening conditions [8]. Therefore, when iGAS is suspected, prompt Multidisciplinary Team (MDT) consultation is required [9]. Based on imaging examinations, the infection scope should be accurately assessed, and anti-infective and/or surgical intervention should be implemented to interrupt the invasive infection pathway [10].

Although iGAS infection and rhinogenic orbital complications have been widely reported, the rapid progression of iGAS to the stage of intraorbital abscess within hours is extremely rare and highly dangerous in the literature [11]. This paper reports a case of a girl whose condition rapidly progressed from the initially diagnosed acute tonsillitis to intraorbital abscess within 6 h, providing important reference for clinically identifying invasive infections with high occult nature and extremely rapid progression, as well as optimizing treatment strategies. This case report aims to enhance pediatricians’ awareness of invasive infections and facilitate timely diagnosis.

## Case presentation

A 6-year-old girl, weighing 26 kg with no history of personal or familial specific diseases, was admitted to the Pediatric Department with the chief complaint of clear rhinorrhea accompanied by cough for 1 week and fever for 2 days. One week prior, the girl developed intermittent, mild clear rhinorrhea. she began to suffer from fever with a maximum body temperature of 39.3℃, along with nasal obstruction, mild cough and lethargy. No vomiting or disturbance of consciousness was noted.

Physical examination on admission revealed the following findings: no eyelid swelling or purulent discharge in either eyes; unrestricted ocular movement, no orbital tenderness, no proptosis, and no limitation of eye opening; no purulent discharge was observed in the nasal cavity, external auditory canals, pharynx or tonsils; no oral herpes was present; and no rales were auscultated in the lungs. Hematological examinations showed: white blood cell count of 31.84 × 10⁹/L, neutrophil ratio of 91.7%, and C-reactive protein (CRP) level of 168.4 mg/L. The pediatrician diagnosed the child with acute tonsillitis and initiated anti-infective therapy. After a negative penicillin skin test, intravenous amoxicillin-clavulanate potassium was administered at a dose of 30 mg/kg every 8 h.

Four hours after admission, the girl developed right eyelid edema and conjunctival hyperemia without discharge, while the left eye appeared normal and her mental status remained poor. A tentative diagnosis of “infectious fever and right conjunctivitis” was made at that time; the aforementioned anti-infective treatment was continued, and moxifloxacin hydrochloride eye drops were added. Two hours later, the girl’s fever persisted, and obvious right eye proptosis. Repeated physical examination showed: poor mental status but clear consciousness; swelling of the right eyelid and periorbital region, with proptosis and tenderness of the right eye and limitation of eye opening; no purulent discharge was noted; the left eye remained normal. Pupillary reflexes: pupil diameter 4.5 mm on the right and 3.0 mm on the left. The right direct pupillary light reflex was sluggish, while the consensual light reflex was preserved. All pupillary reflexes were normal on the left. The girl developed a relative afferent pupillary defect (RAPD) in the right eye. The Department of Pediatrics immediately initiated a MDT consultation.

**Fig. 1:**
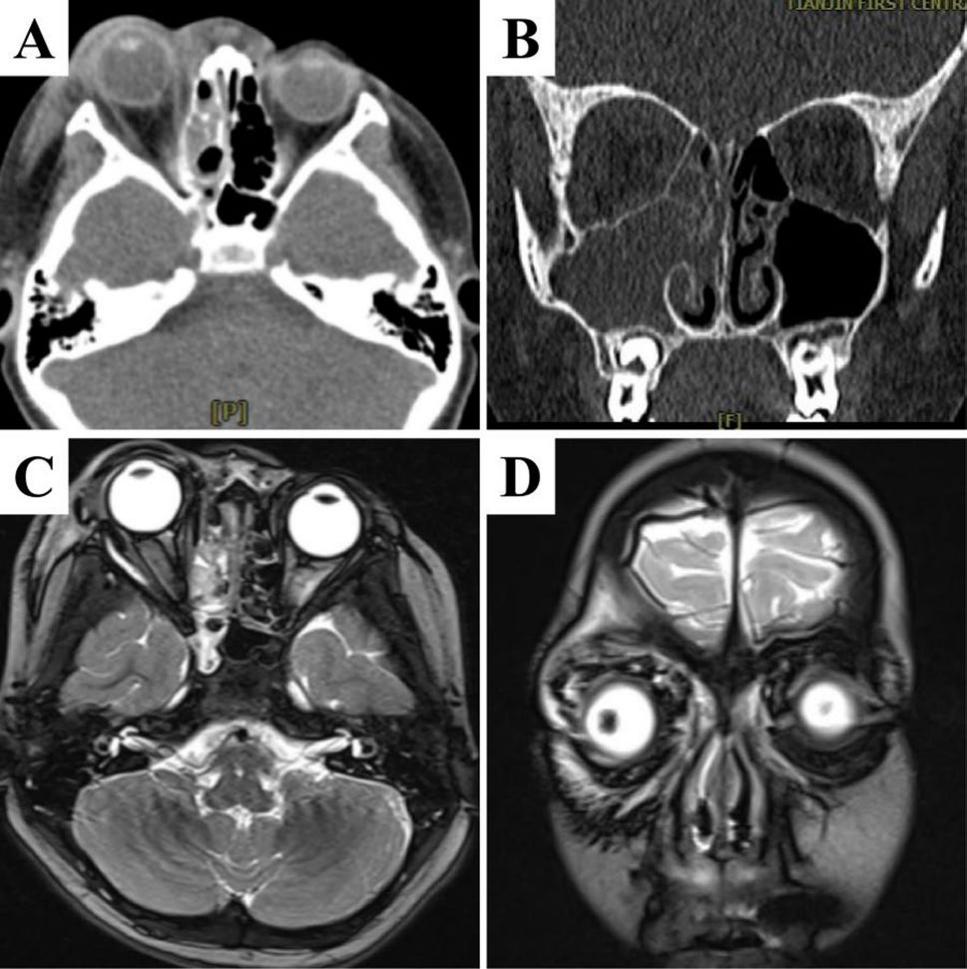
Imaging findings of the patient. **a** and **b** Preoperative orbital CT scans: Periorbital soft tissue swelling with pneumocele, findings consistent with orbital cellulitis. **c** and **d** Preoperative sinus MRI scans: Inflammation is present in the right maxillary sinus, ethmoid sinus, and sphenoid sinus, accompanied by a right orbital abscess

The ophthalmologist suspected acute right orbital cellulitis. Considering the rapid progression of the disease, pediatricians planned to escalate the anti-infective regimen. Specialists from the Department of Infectious Diseases and clinical pharmacists recommended a broad-spectrum antimicrobial coverage strategy, switching to meropenem combined with vancomycin, while continuing the administration of moxifloxacin hydrochloride eye drops. Otolaryngologists advised an immediate orbital computed tomography (CT) scan and paranasal sinus magnetic resonance imaging (MRI). Orbital CT demonstrated periorbital soft tissue swelling and emphysema, suggestive of orbital cellulitis (Fig. 1a and b). Paranasal sinus MRI revealed inflammation in the right maxillary, ethmoid and sphenoid sinuses, together with a right intraorbital abscess (Fig. 1c and d). Subsequent delayed-return results of blood culture and next-generation sequencing (NGS) both definitively identified GAS. Ophthalmological examination showed: visual acuity of 0.3 sc/0.5 sc; intraocular pressure (assessed by digital tonometry) of Tn/15 mmHg; visual field testing yielded no definitive results due to poor cooperation of the child.

**Fig. 2:**
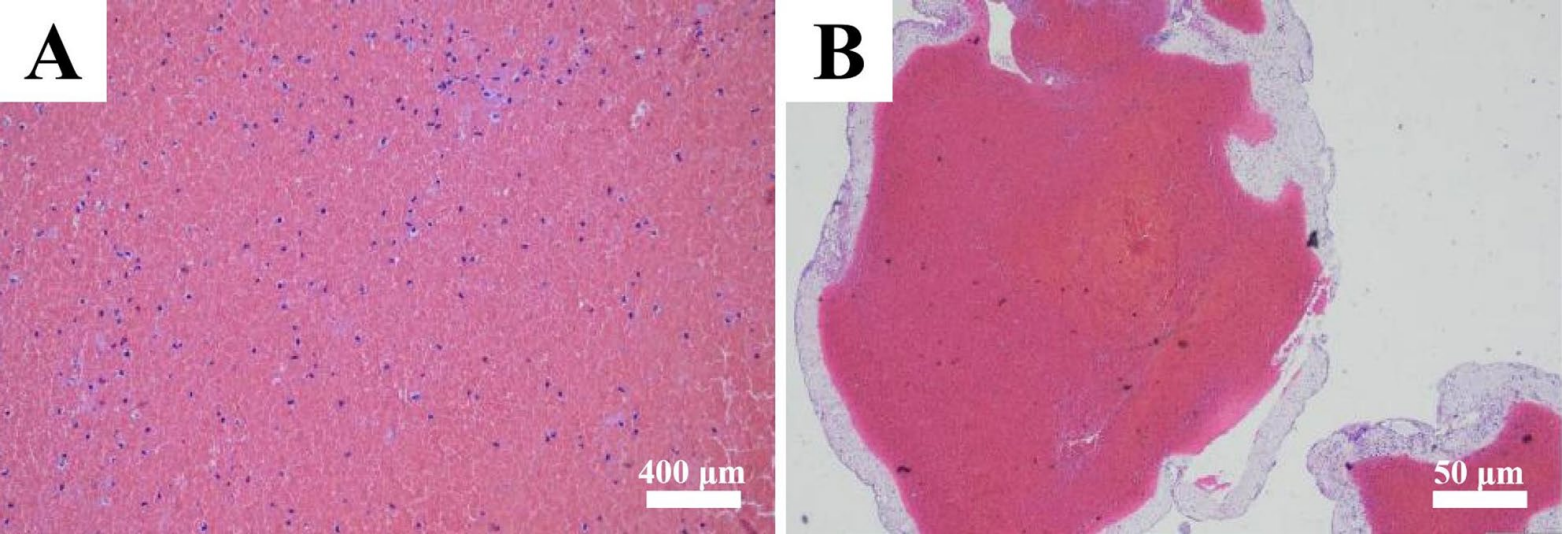
Pathological diagnosis of the thrombus in the right nasal cavity: **a** Magnification at 40×; **b** Magnification at 200×

**Fig. 3:**
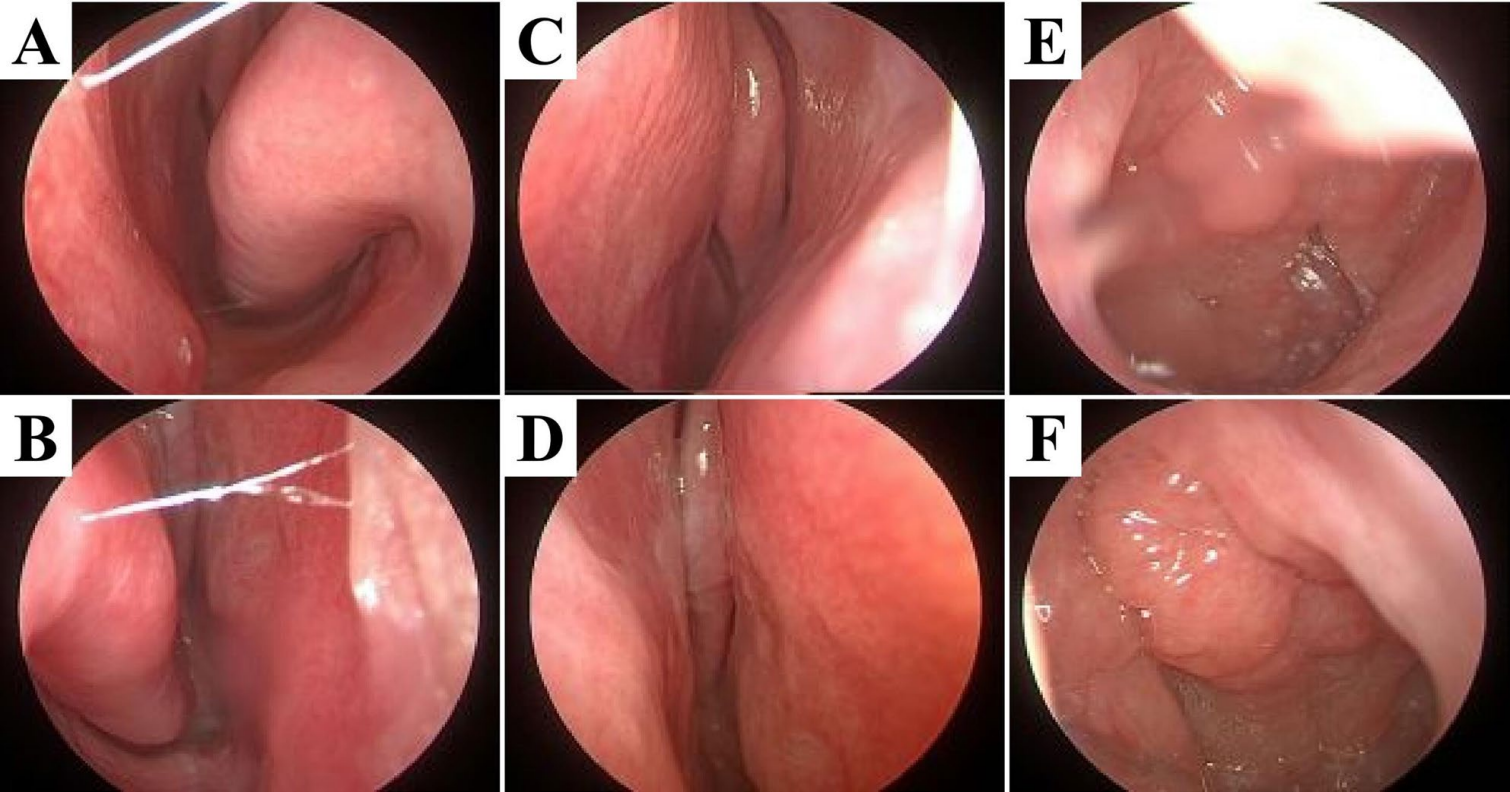
Microscopic findings are as follows: **a**, **b** Bilateral nasal mucosa appeared pale red, nasal septum was roughly in the midline; bilateral inferior turbinates had mild hypertrophy; bilateral middle meatus remained patent with no new masses detected; **c**, **d** No new masses were found in bilateral olfactory clefts; **e**, **f** Mucosal protrusion was observed on the posterior wall of the nasopharynx

Following evaluation by MDT consultation, an emergency surgery was scheduled. The child first received methylprednisolone at a dosage of 1 mg/kg for anti-inflammatory treatment, followed by endoscopic right maxillary and ethmoid sinusotomy combined with right supra-eyebrow approach for intraorbital abscess incision and drainage. Pus culture indicated infection with GAS. Antimicrobial susceptibility testing showed that the isolate was sensitive to amoxicillin-clavulanate. Postoperatively, the anti-infective regimen was formulated collectively by the MDT: amoxicillin-clavulanate potassium was continued. The girl’s proptosis and periorbital swelling resolved gradually. Pathological examination of the surgical specimen indicated a blood clot in the right nasal cavity, ruling out neoplastic diseases (Fig. 2). Anti-infective therapy was maintained for 3 weeks, during which the child’s body temperature stabilized, inflammatory indicators returned to normal, and visual acuity recovered completely. No recurrence was observed during 6-month follow-up period (Fig. 3).

## Discussion and conclusions

This case demonstrates that GAS can stealthily invade the orbit through the fragile medial orbital wall in children, and the disease has already progressed to the high-risk, rapidly progressive stage of intraorbital abscess by the time of definitive diagnosis. It highlights the necessity for the MDT to accurately determine the infection scope and severity based on imaging examinations. GAS is a common pathogenic bacterium causing upper respiratory tract infections in children and is prone to invading the paranasal sinuses [12]. Once it breaches the anatomical barrier of the orbit [13], it will develop into an invasive infection, which then progresses rapidly into periorbital cellulitis characterized by high occultity and an acute clinical course. Due to the non-specificity of ocular symptoms, this condition is highly susceptible to misdiagnosis.

In terms of disease progression, the girl initially presented only with acute tonsillitis but then experienced rapid disease progression. Four hours after admission, eyelid edema developed, followed by the onset of proptosis a further two hours later. The medial wall of the pediatric ethmoid sinus serves as the lateral wall of the nasal cavity, also known as the lamina papyracea, which is extremely thin with a thickness of only 0.2–0.5 mm [14]. However, the bony plate remains incompletely ossified in childhood, with natural small foramina or fissures present (Fig. 1a and c). The MDT concluded that GAS first infected the girl’s upper respiratory tract, then proliferated extensively in the sinus mucosa and eroded the lamina papyracea. Once the bacterium breaches this anatomical barrier, it progresses to an invasive infection [15]. This case was exactly an acute infection caused by iGAS, which led to the involvement of orbital tissues progressing rapidly from orbital cellulitis to the stage of intraorbital abscess within a mere 2 h. This pathological process is characterized by a highly occult early phase, followed by rapid progression after the onset of symptoms. An analysis of pediatric iGAS infection conducted by Barza et al. revealed that the condition progresses extremely rapidly, with a high tendency to develop into necrotizing fasciitis, shock, and multiple organ failure [16]. Furthermore, a consensus has been reached in current literature that approximately 6% of pediatric upper respiratory tract infections progress to acute sinusitis [17–18]. GAS is a common pathogen colonizing the pharynx of children, with an extremely small proportion progressing to iGAS infection. Taking Beijing Children’s Hospital as an example, only 42 cases of iGAS have been documented in published literature over the past decade, among which 1 case progressed from sinusitis to orbital cellulitis [19]. Procacci et al. reported a case of orbital cellulitis and intraorbital abscess secondary to periodontitis, in which the patient’s iGAS rapidly invaded the orbital tissues by breaching the barrier between the roof of the maxillary sinus and the floor of the orbit [20]. Similar reports include a case of purulent meningitis caused by GAS bloodstream infection originating from sinusitis in a 7-year-old child, demonstrating that GAS can penetrate the sinus tissue barrier [21]. Somsen et al. reported a case of orbital cellulitis occurring 1 day after bilateral medial rectus recession in a 6.5-year-old boy, which was attributed to preoperative exposure to a GAS pharyngitis patient [22]. This case suggests that invasive infections progress rapidly and necessitates timely administration of broad-spectrum, potent antibiotics. These studies support the hypothesis proposed in the present case: GAS breaches the lamina papyracea in a highly occult manner, subsequently causes invasive infection of orbital tissues, and progresses rapidly to the stage of intraorbital abscess.

The particularity of this case lies in the fact that the iGAS infection was highly occult in the early phase, yet progressed extremely rapidly once it breached the anatomical barrier, eventually manifesting as observable ocular symptoms. From the initial presentation of the girl, pediatricians paid close attention to the markedly elevated CRP. Although the pathogenic bacteria and infectious focus were not identified at the initial diagnosis stage, empirical anti-infective therapy was promptly initiated, thus avoiding treatment delay.

In terms of diagnosis and treatment, the girl was initially diagnosed with acute tonsillitis, with her CRP level as high as 168.4 mg/L. According to the literature consensus, when CRP is significantly elevated (> 80 mg/L), there is an extremely high risk of developing sepsis, and anti-infective therapy should be promptly initiated [23]. Liao et al. documented a 6-month-old infant presenting with orbital abscess secondary to Staphylococcus aureus infection, accompanied by a markedly elevated CRP level of 147.44 mg/L; the lesion was rapidly and accurately identified via the combined application of CT and MRI [24]. However, when determining the next-step diagnosis and treatment plan, the nature of the infection had not yet been confirmed in this case.

Kurokawa et al. documented a case of iGAS-associated septic shock, where the diagnosis was established using abdominal CT findings before blood culture and NGS results were obtained [25]. The key turning point in the successful diagnosis and treatment of this case was the initiation of MDT consultation, through which the combined application of CT and MRI imaging modalities accurately and rapidly identified the infection sites and pathological characteristics, confirming a diagnosis of right-sided maxillary, ethmoid, and sphenoid sinusitis complicated with right orbital abscess. There is a high probability that such rapidly progressive, special and rare cases will progress to Stage V orbital complications, namely the development of cavernous sinus thrombosis and even intracranial infection [26]. In accordance with the characteristics of the Chandler classification and relevant reports, orbital abscess in the rapidly progressive stage should undergo surgical intervention within 24 h [27]. Meanwhile, the patient developed RAPD, indicating optic nerve involvement that required urgent surgical intervention. Intravenous methylprednisolone was given preoperatively to control inflammation, prevent cytokine storm, reduce local vascular dilation and permeability, and enhance regional tissue perfusion [28]. Subsequently, the patient underwent endoscopically assisted right maxillary and ethmoid sinusotomy, combined with incision and drainage of the right orbital abscess via a supraorbital approach. Considering the potent broad-spectrum antimicrobial activity of the meropenem/vancomycin combination regimen used during the rapid progression phase of the disease, the postoperative anti-infective regimen was adjusted to avoid adverse effects such as the emergence of drug-resistant bacteria, superinfection, and the impact of intestinal flora disruption on the immune system [23]. Postoperatively, the child was treated with amoxicillin-clavulanate potassium for anti-infective therapy over a 3-week period and remained recurrence-free during 6 months of follow-up, which further confirmed the previous conclusion that GAS rapidly invaded the orbital tissues by breaching the lamina papyracea barrier.

The main limitation of this case report is that blood culture results were not yet available during the rapidly progressive phase of the disease; therefore, the diagnosis of infection with highly occult iGAS was based on accurate comprehensive assessment via MDT and multiple indirect lines of evidence, including markedly elevated CRP levels in the girl, failure of conventional anti-infective therapy, and rapid progression of ocular symptoms. The accurate diagnosis and treatment of this case was achieved mainly by virtue of the meticulous collection of the girl’s symptoms and signs by pediatricians, the decisive management of the rapidly progressive condition, and the timely initiation of MDT consultation.

In conclusion, iGAS infection is a rare etiology of rhinogenic orbital abscess, owing to its highly occult breach of the orbital anatomical barrier. This condition is characterized by a high degree of occultism. And by the time of clinical diagnosis, it has already progressed to the rapidly progressive stage of intraorbital abscess. In this case, the abnormal ocular signs of the girl were highly valued by pediatricians, and the MDT consultation was decisively initiated. Through individualized anti-infective therapy and precise surgical treatment, the goals of early inflammation control, rapid lesion eradication, and consolidation of anti-infective therapy were effectively achieved. This case enriches the phenotypic spectrum of severe iGAS infection and provides valuable reference for the individualized clinical diagnosis and treatment of associated severe orbital complications.

## Data Availability

The datasets used and/or analysed during the current study are available from the corresponding author on reasonable request.

## Abbreviations

iGAS: Invasive Group A Streptococcal
CRP: C-reactive Protein
MDT: Multidisciplinary Team
RAPD: Relative Afferent Pupillary Defect
CT: Computed Tomography
MRI: Magnetic Resonance Imaging
NGS: Next-Generation Sequencing

## References

[1] Sherwood E, Vergnano S, Kakuchi I, et al. Invasive group A streptococcal disease in pregnant women and young children: a systematic review and meta-analysis. Lancet Infect Dis. 2022;22(7):1076–88. 35390294 10.1016/S1473-3099(21)00672-1 PMC9217756

[2] Cole JN, Barnett TC, Nizet V, et al. Molecular insight into invasive group A streptococcal disease. Nat Rev Microbiol. 2011;9(10):724–36. 21921933 10.1038/nrmicro2648

[3] Jiang L, Jiang X, Wu W, et al. Occult medial orbital wall trapdoor fracture inducing recurrent rhinogenic intraorbital abscess in a pediatric patient: a case report. BMC pediatr. 2025;25(1):942. 41272552 10.1186/s12887-025-06331-y PMC12639652

[4] Salgado-Lopez L, Leonel L, C P C, O’brien M, et al. Anatomical step-by-step dissection of complex skull base approaches for trainees: endoscopic endonasal approach to the orbit. J Neurol Surg Part B. 2023;84(01):79–88. 10.1055/a-1723-1675 PMC9897906 36743715

[5] Deniz MA, Tekinhatun M. Evaluation of lamina papyracea dehiscence with paranasal computed tomography. Eur Arch Oto-Rhino-L. 2024;281(7):3649–54. 10.1007/s00405-024-08538-8 PMC11211170 38466422

[6] Yoshida H, Kihara C, Satoh C, et al. CT analysis of predictors for visual acuity in optic neuropathy with mucocele. Auris Nasus Larynx. 2023;50(6):895–903. 36967263 10.1016/j.anl.2023.03.003

[7] Chandler JR, Langenbrunner DJ, Stevens ER. The pathogenesis of orbital complications in acute sinusitis. Laryngoscope. 1970;80(9):1414–28. 5470225 10.1288/00005537-197009000-00007

[8] Anselmo-Lima WT, Soares MR, Fonseca JP, et al. Revisiting the orbital complications of acute rhinosinusitis. Braz J Otorhinolar. 2023;89:101316. 10.1016/j.bjorl.2023.101316 PMC10495636 37678009

[9] Taylor A, Elliott BM, Atkinson J, et al. Group A Streptococcus primary peritonitis in children, New Zealand. Emerg Infect Dis. 2023;29(11):2203–9. 37878292 10.3201/eid2911.230211 PMC10617357

[10] Gregory CJ, Okaro JO, Reingold A, et al. Invasive group A streptococcal infections in 10 US states. JAMA. 2025;333(17):1498–507. 40193120 10.1001/jama.2025.0910 PMC11976646

[11] Vloka CN, Kim DH, Ng JD. Microbiology of orbital cellulitis with subperiosteal abscess in children: prevalence and characteristics of Streptococcus anginosus group infection. Orbit. 2022;41(2):204–10. 33386062 10.1080/01676830.2020.1862247

[12] Haug RH. Microorganisms of the nose and paranasal sinuses. Oral Maxil Surg Clin. 2012;24(2):191–6. 10.1016/j.coms.2012.01.001 22341509

[13] Cornelius CP, Mayer P, Ehrenfeld M, et al. The orbits-Anatomical features in view of innovative surgical methods. Facial Plast Surg. 2014;30(05):487–508. 25397705 10.1055/s-0034-1394303

[14] Açar G, Büyükmumcu M, Güler İ. Computed tomography based analysis of the lamina papyracea variations and morphology of the orbit concerning endoscopic surgical approaches. Braz J Otorhinolar. 2019;85(5):551–9. 10.1016/j.bjorl.2018.04.008 PMC9443057 29859679

[15] Barza M. Anatomical barriers for antimicrobial agents. Eur J Clin Microbiol. 1993;12(1):S31–5. 10.1007/BF02389875 8477760

[16] Mercadante S, Ficari A, Romani L, et al. The thousand faces of invasive group A streptococcal infections: update on epidemiology, symptoms, and therapy. Children-Basel. 2024;11(4):383. 38671600 10.3390/children11040383 PMC11048970

[17] Wald ER, Applegate KE, Bordley C, et al. Clinical practice guideline for the diagnosis and management of acute bacterial sinusitis in children aged 1 to 18 years. Pediatrics. 2013;132(1):e262–80. 23796742 10.1542/peds.2013-1071

[18] Leung AKC, Hon KL, Chu WCW. Acute bacterial sinusitis in children: an updated review. Drugs Context. 2020;9:2020–9. 33281908 10.7573/dic.2020-9-3 PMC7685231

[19] Liu B, Liu G, Qian S, et al. Clinical features of invasive Group A Streptococcus infection in children. Chin J Pract Pediatr. 2025;40(12):927–32.

[20] Procacci P, Zangani A, Rossetto A, et al. Odontogenic orbital abscess: a case report and review of literature. Oral Maxillofac Surg. 2017;21(2):271–9. 28303354 10.1007/s10006-017-0618-1

[21] Lee J, Blackburn J, Pham-Huy A. Uncommon clinical presentation of a common bug: Group A Streptococcus meningitis. Pediatr Child Health-Can. 2021;26(3):e129–31. 10.1093/pch/pxaa065 PMC8077209 33936341

[22] Somsen D, Heidary G. Rapid onset of orbital cellulitis after uncomplicated strabismus surgery. J AAPOS. 2019;23(5):290–1. 31185285 10.1016/j.jaapos.2019.05.006

[23] Jung C, Levy C, Béchet S, et al. Impact of C-reactive protein point-of-care testing on antibiotic prescriptions for children and adults with suspected respiratory tract infections in primary care: a French patient-level randomized controlled superiority trial. Clin Microbiol Infec. 2024;30(12):1553–8. 39067513 10.1016/j.cmi.2024.07.014

[24] Liao D, Shi B, Zhu Y. Drainage of intraorbital abscess in infant via endoscopic nasalsinus approach: a case report. Chin J Otorhinolaryngol Head Neck Surg. 2021;56(11):1204–6. 10.3760/cma.j.cn115330-20201223-00946 34749461

[25] Kurokawa R, Kurokawa M, Onoda S, et al. Early Diagnosis of Streptococcal Toxic Shock Syndrome With Abdominal Computed Tomography: A Case Report. Cureus. 2025;17(8):e90348. 40970022 10.7759/cureus.90348 PMC12441656

[26] Caranfa JT, Yoon MK. Septic cavernous sinus thrombosis: a review. Surv ophthalmol. 2021;66(6):1021–30. 33831391 10.1016/j.survophthal.2021.03.009

[27] Pereira FJ, Cruz AAV, Anselmo-Lima WT, et al. Computed tomographic patterns of orbital cellulitis due to sinusitis. Arq Bras Oftalmol. 2006;69:513–8. 17119723 10.1590/s0004-27492006000400011

[28] Olsen AA, Strandby RB, Johansson PI, et al. Evaluation of the systemic inflammatory response, endothelial cell dysfunction, and postoperative morbidity in patients, receiving perioperative corticosteroid, developing severe mesenteric traction syndrome-an exploratory study. Langenbecks Arch Surg. 2022;407(5):2095–103. 35397681 10.1007/s00423-022-02507-7

